# Treatment patterns and outcomes of tyrosine kinase inhibitors in chronic myeloid leukemia: a single center study from Oman

**DOI:** 10.3389/fonc.2026.1866929

**Published:** 2026-07-15

**Authors:** Noor Al-Rawahi, Bushra Salman, Dhanalekshmi Unnikrishnan Meenakshi, Murtadha Al-Khabori, Mohammed Al-Hanini, Salam Al-Kindi

**Affiliations:** 1 Pharmacy Department, University Medical City, Muscat, Oman; 2 Department of Pharmacy, National University of Science and Technology, Muscat, Oman; 3 University Medical City, Muscat, Oman; 4 Department of Hematology, Sultan Qaboos University, Muscat, Oman

**Keywords:** chronic myeloid leukemia, imatinib, major molecular response, treatment-free remission, tyrosine kinase inhibitors

## Abstract

**Background:**

Real-world data on tyrosine kinase inhibitor (TKI) use in chronic myeloid leukemia (CML) from the Middle East remain limited. We evaluated treatment patterns, molecular responses, treatment-free remission (TFR), and safety among Omani patients with chronic-phase CML.

**Methods:**

In this retrospective single-center study, adult patients (≥18 years) with chronic-phase CML treated with TKIs between January 2008 and September 2024 were included. Demographics, Sokal risk, treatment lines, molecular responses, TFR, and adverse drug reactions (ADRs) were analyzed.

**Results:**

Among 112 patients (mean age 43.7 ± 15.4 years), 45% had low Sokal risk, 43% intermediate, and 12% high risk. Imatinib was first-line therapy in 88.4%, achieving major molecular response (MMR) in 51%, with a median time to MMR of 21 months. In contrast, second-line TKIs demonstrated higher efficacy, with MMR rates of 79.2% for nilotinib and 54.4% for dasatinib, and shorter time to response (12 vs 23.2 months, respectively). Successful TFR was maintained in 17% of imatinib-treated patients after two years and in 83.3% of second-line patients after 36 months. ADRs were mainly gastrointestinal (28.5%), musculoskeletal (28.0%), dermatologic (20.8%), and hematologic (8.7%). Grade 3–4 events occurred in 5%, leading to TKI switching. Discontinuations were primarily due to loss of response, ADRs, or planned TFR.

**Conclusion:**

In this real-world cohort, first-line imatinib provided acceptable but slower and less frequent molecular responses compared with second-generation TKIs. Higher TFR maintenance rates were observed with second-generation TKIs, although interpretation is limited by small patient numbers. These findings support a response-guided, risk-adapted approach to TKI selection and highlight the need to expand access to second-generation TKIs to optimize long-term outcomes in CML.

## 1 Introduction

Chronic myeloid leukemia (CML) is a myeloproliferative neoplasm defined by the Philadelphia chromosome; a reciprocal translocation between chromosomes 9 and 22 that generates the BCR-ABL1 fusion gene encoding a constitutively active tyrosine kinase (1).

The introduction of tyrosine kinase inhibitors (TKIs), beginning with imatinib in 1998, transformed CML from a fatal disease into a chronic condition with near-normal life expectancy. Imatinib remains a standard frontline therapy, achieving overall response rates of 70–80% and 83% overall survival at 8 years in the IRIS trial, (2). However, 20–30% of patients develop resistance or intolerance, including gastrointestinal, dermatologic, hematologic, and cardiovascular toxicities (3).

Second-generation TKIs (dasatinib, nilotinib, bosutinib) provide faster and deeper molecular responses and are widely used in frontline or resistant settings, though each has distinct toxicity profiles (4). Ponatinib, a third generation TKI, overcomes T315I-mediated resistance, and asciminib, an allosteric ABL1 inhibitor, has demonstrated superior molecular responses compared with imatinib, and it is approved for patients resistant or intolerant to TKIs (5, 6). Selected patients achieving sustained deep molecular response may attempt treatment-free remission (TFR) under careful monitoring (7).

Despite these advances, real-world data (RWD) describing TKI use and outcomes in the Middle East remain scarce. A Turkish study of 106 patients with chronic-phase CML treated with second-generation TKIs reported high initial response rates, though nearly half required treatment switching due to intolerance or resistance (8). Similarly, a survey from the Gulf region highlighted variability in treatment selection, monitoring practices, and financial barriers, with imatinib being the predominant frontline therapy despite international availability of newer agents (9).

Given these gaps, this study aims to evaluate the characteristics, management patterns, adverse drug reactions (ADRs), and outcomes of patients with CML receiving TKI therapy in Oman. Specifically, it seeks to assess real-world patterns of TKI use in chronic-phase CML, including frontline and subsequent lines of therapy, evaluate the safety and efficacy of different TKI generations, characterize the type and frequency of ADRs and their clinical management strategies, assess the rate and success of TFR, and determine the rate and underlying reasons for TKI discontinuation. Findings from this study will provide valuable insights into TKI utilization in an underrepresented population and support alignment of local clinical practice with international CML management standards.

## 2 Materials and methods

### 2.1 Design and setting

This was a single-center retrospective cohort study conducted at Sultan Qaboos University Hospital (SQUH), a tertiary academic medical center in Muscat, Oman. Data were extracted from the hospital’s electronic medical records for patients treated between January 2008 and September 2024. The study was approved by the Medical Research Ethics Committee, College of Medicine and Health Sciences, Sultan Qaboos University, on 17 February 2025 (Ref. SQU-EC/010/2025, MREC #3515).

### 2.2 Participants; inclusion and exclusion criteria

The study included 112 adult Omani patients, 18 years and older, who were diagnosed with chronic-phase CML and treated with at least one line of TKI therapy, including imatinib, nilotinib, dasatinib, ponatinib, or asciminib. Patients with accelerated-phase or blast-phase CML, incomplete medical records, or those who had received TKIs for other indications were excluded.

### 2.3 Data collection

Data were collected using a standardized form and included demographic variables such as age, sex, body mass index, smoking status, and comorbidity burden assessed by the Charlson Comorbidity Index (CCI). Baseline diagnostic parameters included complete blood count (CBC), spleen size, and cytogenetic and molecular analyses such as fluorescence *in situ* hybridization, bone marrow karyotyping, and quantitative PCR for BCR-ABL1 transcript levels. Sokal risk scores were calculated for each patient at diagnosis using the original Sokal model based on age, spleen size documented on physical examination at diagnosis, platelet count, and peripheral blood blast percentage. Treatment data encompassed prior and current TKI regimens, dose modifications, treatment duration, and switching to alternative TKIs.

### 2.4 Outcome definition

Efficacy outcomes were evaluated based on achievement of molecular milestones, particularly major molecular response (MMR), as recommended by the European LeukemiaNet (ELN) 2020 guidelines. Duration until first MMR was categorized into predefined 3-monthly time intervals beginning from <3 months to >36 months to assess treatment response kinetics. Patients attempting TFR met the ELN eligibility criteria for TKI discontinuation. Molecular monitoring was performed according to the institutional practice and ELN recommendation. Molecular relapse was defined as a loss of MMR (BCR::ABL1>0.1% IS)and TKI therapy was resumed at this point. All eligible patients had their TFR milestones and duration recorded. Safety outcomes included TKI-related ADRs, which were graded using the Common Terminology Criteria for Adverse Events (CTCAE) version 5.0. For each patient, the type and severity of ADRs were recorded, along with clinical management strategies such as dose modification, discontinuation, or switching.

Treatment decisions, including the selection and sequencing of TKIs, were at the discretion of the treating hematologist and guided by FDA-approved label indications, clinical judgment, comorbidities, and treatment tolerance. A new line of therapy was defined as initiation of a different TKI following discontinuation of a prior agent due to resistance, intolerance, or suboptimal response.

### 2.5 Sample size and power calculation

No sampling was performed; the study represents a census of all eligible patients during the study period.

An *a priori* sample size estimation based on reported imatinib response rates of approximately 70–80% (IRIS trial) indicated that at least 94 patients would be required to estimate response proportions with a 95% confidence level and 5% margin of error. The final sample comprised 112 patients, exceeding this threshold and providing an estimated statistical power of approximately 87% for detecting response rates within the expected range.

### 2.6 Statistical analysis

Categorical variables were summarized as frequencies and percentages, while continuous variables were reported as means with standard deviations for normally distributed data, or medians with interquartile ranges for skewed data. Associations between categorical variables were tested using Chi-square or Fisher’s exact test, while continuous variables were compared using independent t-tests or Mann–Whitney U tests as appropriate. Comparisons across more than two groups were performed using the Kruskal–Wallis test. All statistical analyses were two-tailed, with a significance threshold of p < 0.05, and conducted using SPSS Statistics version 20 (IBM Corp., Armonk, NY, USA).

## 3 Results

### 3.1 Demographic and clinical characteristics

A total of 112 patients with chronic-phase CML were included. The mean age at diagnosis was 43.7 years (SD 15.4; range 18–80), and 56.3% were female. Nearly one-third of patients were overweight (28.6%) and 26.8% were obese. Only 4.5% of patients were smokers and one reported alcohol use. Sokal risk score was calculated for 107 of the 112 patients, 48 (42.8%) were low risk, 46 (41.1%) were intermediate risk and 13 (11.6%) were high risk. The score could not be calculated for the other five patients (4.5%) due to missing baseline data.

Comorbidity burden was modest, with 29.5% having a CCI score of 1–2, and 16.1% with scores of ≥5. More than half of patients (55.1%) had intermediate or high Sokal risk at diagnosis, while the remainder were classified as low risk. Six women (9.5% of females) experienced pregnancy during the treatment course. Baseline demographic and clinical features are presented in **Table 1**.

**Table 1:** Baseline demographic and clinical characteristics. .

Characteristic N=112	Categories	Frequency (%) (unless otherwise specified)
Gender	Female	63 (56.3)
	Male	49 (43.8)
Age (years), mean ± SD (range)		43.66 ± 15.41 (18-80)
BMI groups*	Underweight	7 (6.3)
	Normal weight	43 (38.4)
	Overweight	32 (28.6)
	Obese class 1	16 (14.3)
	Obese class 2	8 (7.1)
	Obese class 3	6 (5.4)
Sokal score	Low risk	48 (42.8)
	Intermediate risk	46 (41.1)
	High risk	13 (11.6)
	Missing	5 (4.5)
Smoking status	No	107 (95.5)
	Yes	5 (4.5)
Alcohol intake	No	111 (99.1)
	Yes	1 (0.9)
Charlson co-morbidity index (n= 107)	1-2	61 (54.5)
	3-4	33 (29.5)
	> 5	18 (16.1)
Sokal score (n =107)	Low risk	48 (44.9)
	Intermediate risk	46 (43)
	High risk	13 (12.1)

BMI, body mass index, SD, standard deviation.

*BMI categories: underweight (<18.5 kg/m^2^); normal weight (18.5-24.9 kg/m^2^); overweight (25.0-29.9 kg/m^2^); obese class1 (30.0 -34.9 kg/m^2^); obese class 2 (35.0- 39.9 kg/m^2^); obese class 3 (≥ 40.0 kg/m^2^).

### 3.2 Treatment patterns

All patients received at least one line of TKI therapy. Imatinib was the most common first-line treatment (88.4%), followed by nilotinib (8.9%). Two patients received asciminib as first-line therapy through a manufacturer-sponsored patient access program and was analyzed according to the treatment received. Second-line therapy was required in 50 (44.6%) of patients, most frequently with nilotinib [48% (24/50)] or dasatinib [44% (22/50)]. Seventeen patients (15.2%) received third-line treatment, most commonly nilotinib [29.4% (5/17)] or imatinib [23.5% (4/17)]. Five patients proceeded to a fourth-line regimen, and only one required fifth-line therapy with asciminib. First-line, second-line and third-line TKI therapy has median duration of 42.7, 41.7 and 7.6 months, respectively. The median duration between diagnosis and the start of second line and third line TKI treatment was 25.9 and 51.3 months, respectively. The relatively shorter duration of third-line therapy reflects the recent initiation of treatment in several patients and the study data cutoff **Table 2** shows TKI treatment patterns at different therapy lines.

**Table 2:** Treatment patterns of TKI medications in the study sample.

TKI treatment line	Drug	N (%)
1^st^ line (n =112)	Imatinib	99 (88.4)
	Nilotinib	10 (8.9)
	Dasatinib	1 (0.9)
	Ponatinib	0 (0)
	Asciminib	2 (1.8)
Switched to the 2^nd^ line (n =112)	Yes	50 (44.6)
	No	62 (55.4)
2^nd^ line (n =50)	Imatinib	1 (2.0)
	Nilotinib	24 (48)
	Dasatinib	22 (44)
	Ponatinib	2 (4)
	Asciminib	1 (2)
Switched to the 3^rd^ line (n =50)	Yes	17 (34)
	No	33 (66)
3^rd^ line (n =17)	Imatinib	4 (23.5)
	Nilotinib	5 (29.4)
	Dasatinib	3 (17.6)
	Ponatinib	3 (17.6)
	Asciminib	2 (11.8)
Switched to the 4^th^ line (n =17)	Yes	5 (29.5)
	No	12 (70.5)
4^th^ line (n =5)	Imatinib	3 (60)
	Nilotinib	2 (40)
Switched to the 5^th^ line (n =5)	Yes	1 (20)
	No	4 (80)
5^th^ line (n =1)	Asciminib	1 (100)

TKI, tyrosine kinase inhibitor.

**Figure 1:**
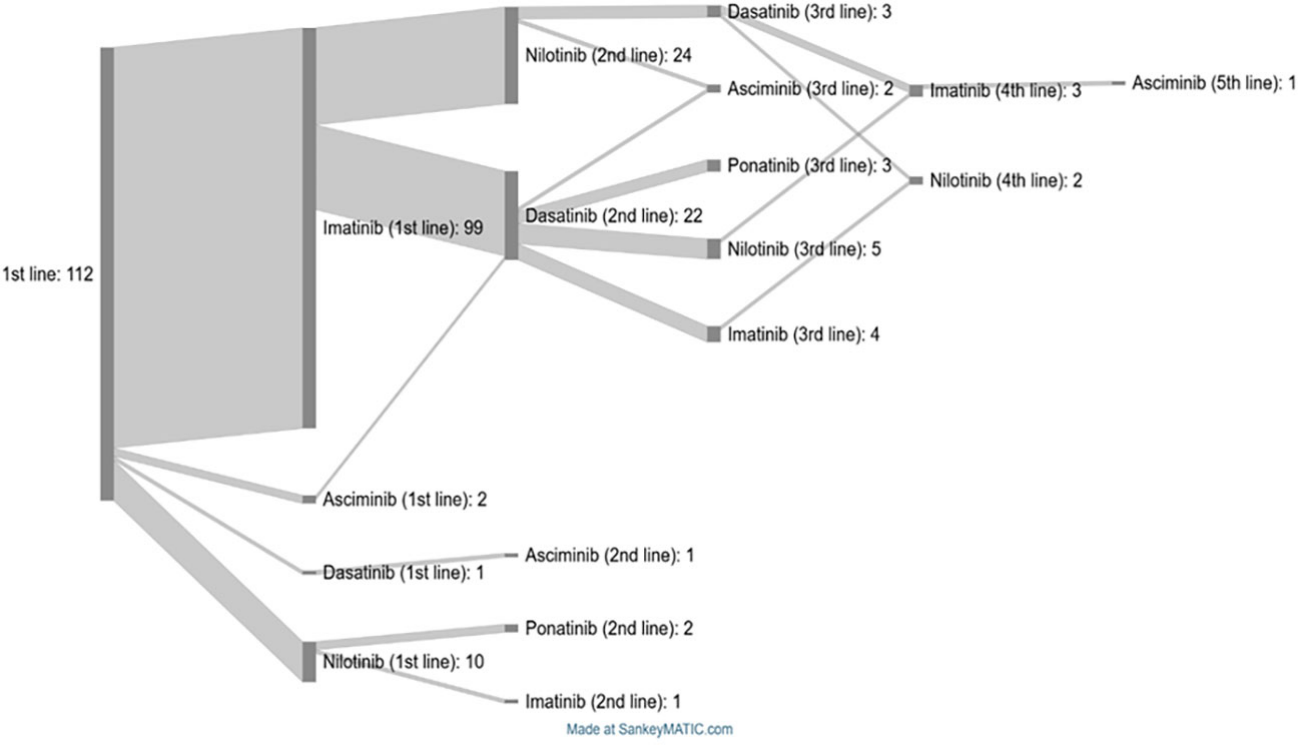
Sankey diagram of detailed medication switching patterns.

Overall, 55.4% of patients remained on first-line therapy without switching, while 29.4% received two lines, 10% three lines, and 4.5% four or more lines. Treatment sequencing was heterogeneous: among the 99 patients who started on imatinib, 50 switched to a second-line agent. Imatinib was used in certain patients as a second- or third-line treatment after a second generation TKI, primarily because of intolerance to prior therapy, patient-specific clinical considerations and/or limitations in drug availability. TKI cycling was observed in four patients, all of whom received imatinib as first-line therapy, switched to dasatinib in second-line setting, and subsequently returned to imatinib as third-line therapy. Cycling was primarily driven by intolerance, ADRs and treatment availability. Patients initially treated with nilotinib were more likely to switch to asciminib or dasatinib, while those on dasatinib moved to ponatinib, nilotinib, or imatinib. In patients with treatment failure requiring second-line or subsequent TKI therapy, mutational analysis, including assessment for T315I mutation, was performed in the majority of cases to guide treatment selection. One patient was identified with a T315I mutation and subsequently underwent bone marrow transplant. As patients could receive multiple sequential TKIs during the study period, the number of treatment episodes exceeds the number of unique patients included in the cohort. Treatment transitions across successive lines of therapy are illustrated in the Sankey diagram (**Figure 1**).

### 3.3 Achievement of MMR

In the overall cohort, 50% of patients (56/112) achieved MMR on first-line TKI therapy. The median time to MMR was 18.7 months (95% CI: 11 – 26.4). Most responses occurred late: 33.9% achieved MMR after 36 months, while 19.6% achieved between 12–18 months; no patient achieved MMR within the first three months. Among responders, 30.4% (17/56) subsequently lost MMR.

**Figure 2:**
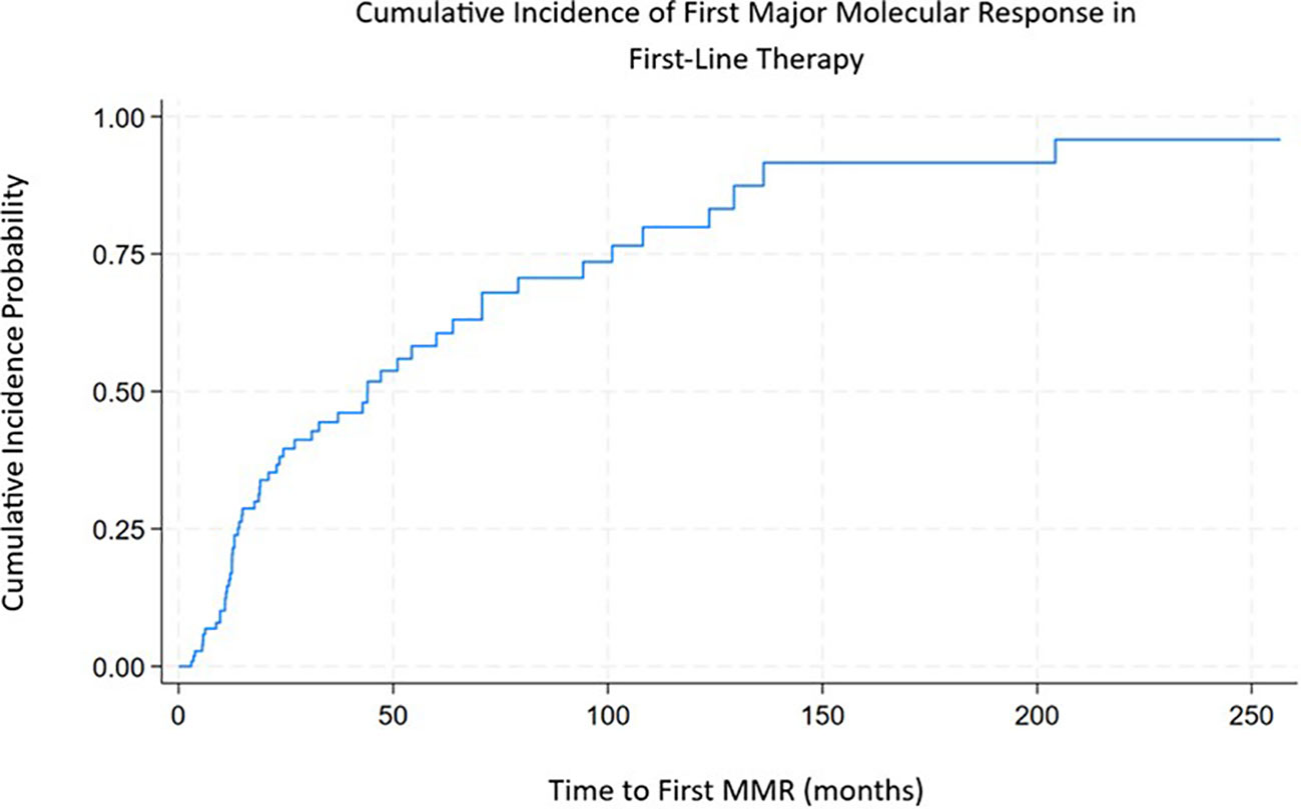
Cumulative incidence of first major molecular response (MMR) among patients receiving first-line TKI therapy.

By first-line treatment, MMR was achieved in 51.5% (51/99) of patients treated with imatinib and 50% (5/10) of those receiving nilotinib. Median time to MMR was longer with imatinib (21 months) compared with nilotinib (5.5 months). Four of five patients treated with nilotinib achieved MMR within 3–6 months. No MMR was observed among patients treated with first-line dasatinib (n = 1) or asciminib (n = 2); however, the very small number of patients receiving these agents precludes meaningful interpretation of treatment outcomes. Loss of MMR occurred in 31.4% (16/51) of imatinib responders and 20% (1/5) of nilotinib responders (p = 0.99). **Figure 2** illustrates overall time to MMR for first-line TKI cohort.

In the second-line setting, MMR was achieved in 19/24 (79.2%) of patients treated with nilotinib, 12/22 (54.5%) with dasatinib, and 2/2 (100%) patients treated with ponatinib. No MMR was observed among patients receiving second-line imatinib (n = 1) or asciminib (n = 1), although patient numbers in these subgroups were small. Differences in cumulative incidence approached but did not reach statistical significance (p = 0.055). Median time to MMR was 12 months (95% CI: 5.1–19.3) with nilotinib and 23.2 months (95% CI: 3.6–50.0) with dasatinib.

MMR loss occurred in 31.6% (6/19) of nilotinib responders and 50% (6/12) of dasatinib responders, but in none of those treated with ponatinib (p=0.427).

In the third-line setting, only 2 of 17 patients (11.8%) achieved MMR: one on imatinib (after 5.3 months, sustained) and one on dasatinib (after 3.2 months, lost subsequently).

Baseline characteristics, including age, white blood cell count, platelet count, hemoglobin, sex, and BMI, were compared between patients who achieved MMR and those who did not. No statistically significant differences were observed between the two groups for any of the variables examined (all p > 0.05).

Following this, baseline sokal risk category was evaluated in relation to MMR achievement. No significant association was observed in the first-line therapy (p-value = 0.170) or third-line (p-value =1.0). In second-line setting, MMR rates were 94.1% (16/17), 71.4% (10/14) and 50.0% (4/8) among low, intermediate and high Sokal risk patients, respectively, demonstrating a significant association between lower Sokal risk and MMR achievement (p-value=0.032).

### 3.4 Achievement of TFR

Throughout several lines of TKIs therapy, a total of 26 patients attempted TFR. Out of 112, 19 patients (17%) in the first-line therapy became eligible to attempt TFR; all of these patients received imatinib. The majority of these individuals, who ranged in age from 26 to 64 (mean age 38.6 years) and had a CCI 2-3. Notably, in all the patients in this group, TFR was attempted after more than 36 months. The median length of TFR was 45.4 months with a sustained TFR seen in ~65% of the patients. Among patients who lost TFR, molecular relapse occurred 104, 106, 115, 151, 167, 210, and 5482 days after TKI discontinuation. At the time of analysis, 12 patients remained in TFR. Of these, 10 had maintained TFR for at least 36 months, while 2 had shorter follow-up durations (<36 months) but remained in TFR at their most recent assessment. TFR outcomes of first-line TKIs are shown in **Table 3**.

**Table 3:** TFR measures of the 1^st^ line TKI in the study cohort.

Efficacy measure	Category	n	%
TFR attempted (n =112)	Yes	19	17.0
	No	93	83.0
Duration of TKI therapy before TFR attempt (months)	> = 36	19	100.0
Duration (months) of TFR at last follow-up (n =19)	< 3 months	1	5.26
	3 – 6	5	26.3
	6 – 9	1	5.26
	12 – 18	1	5.26
	24 – 36	1	5.26
	> = 36	10	52.6
Loss of TFR (n =19)	Yes	7	36.8
	No	12	63.2

TFR, treatment-free remission.

In the second line, 6/50 patients (12%) became eligible to attempt TFR, after switching to dasatinib and nilotinib. Patient profiles remained consistent with a predominantly low comorbidity burden. All of the patients in this group attempted TFR after more than 36 months of treatment, with the exception of one patient who attempted TFR shortly after completing 12 months of dasatinib therapy following a switch from previous TKI. The median TFR duration was approximately 20 months and five of six patients (83.3%) maintained TFR, with only one patient experiencing molecular relapse after three months.

In the third line therapy setting, only 1 out of 17 patients (5.9%) became eligible to attempt TFR on nilotinib, after more than 36 months of therapy and remained in remission at last follow-up. This case similarly reflected a low comorbidity profile, with a TFR duration of ~63 months.

### 3.5 Safety endpoints

Concerning safety outcomes, a total of 183 TKI-related ADRs were reported across the cohort., presented in **Table 4**. Because TKI utilization differed substantially across treatment groups, ADRs were evaluated relative to drug exposure. Although imatinib accounted for 62% of all reported ADRs, it was also the most frequently prescribed TKI, being used in 88.4% of patients as first-line therapy. Nilotinib and dasatinib accounted for 19.0% and 14.6% of ADRs, respectively, while only three ADRs each were reported with ponatinib and asciminib. Overall, gastrointestinal (29%) and musculoskeletal (28%) toxicities were the most common ADR categories, followed by dermatologic events (20.8%). Hematologic, cardiovascular/neurological, and genitourinary toxicities were less frequently observed.

**Table 4:** Adverse Drug Reactions according to TKI exposure.

TKI	ADR category	ADR grades count			
		1	2	3	4
Imatinib					
Gastrointestinal		7	27	–	–
Cardiovascular and/or Neurological		1	5	–	–
Musculoskeletal		12	28	–	–
Hematological		3	1	4	–
Ophthalmology		2	1	–	–
Dermatological		5	15	–	–
Genitourinary		1	2	–	–
Nilotinib					
Musculoskeletal		2	5	–	–
Gastrointestinal		3	7	–	–
Dermatological		4	5	–	–
Cardiovascular and Neurological		2	3	–	–
Hematological		1	–	1	1
Genitourinary		–	1	–	–
Dasatinib					
Gastrointestinal		1	7	1	–
Cardiovascular and Neurological		–	3	1	–
Hematological		1	1	1	–
Dermatological		5	1	–	–
Musculoskeletal		–	4	–	–
Genitourinary		–	1	–	–
Ponatinib					
Dermatological		1	–	1	–
Hematological		–	–	1	–
Asciminib					
Dermatological		–	1	–	–
Ophthalmology		1	–	–	–
Hematological		1	–	–	–

ADR, Adverse Drug Reaction.

According to CTCAE grading, most ADRs were of mild to moderate severity, with 65% classified as grade 2 and 29% as grade 1. Of the 10 grade 3 ADRs reported, seven were hematological, including pancytopenia, neutropenia, and leukocytosis. These events were managed through treatment interruption, switching to an alternative TKI, administration of G-CSF (filgrastim) when indicated, and close monitoring of blood counts.

The only hematological grade 4 ADR reported with nilotinib was managed by discontinuing of the TKI and switching to another; most of the grade 1 ADRs were handled by monitoring CBC levels with iron replacement for anemia, as appropriate.

As presented in **Table 4**, Imatinib 400 mg demonstrated the highest burden of toxicity across all doses, where 24 musculoskeletal ADRs (e.g., cramps, pain), 24 gastrointestinal ADRs (e.g., nausea, gastritis, vomiting), and 13 dermatological ADRs were documented. The majority (97%) were grade 1–2, with rare grade 3 neutropenia events. ADRs at other doses were fewer, with gastrointestinal symptoms being the most consistent finding.

Nilotinib was associated with fewer ADRs overall, though higher doses (600 mg) resulted in 16 ADRs, including gastrointestinal, cardiovascular, and neurological events. Dasatinib toxicity was dominated by gastrointestinal and dermatological events. At the 100 mg dose, 21 ADRs were reported, mostly mild rash and itching, with occasional musculoskeletal ADRs. Notably, dasatinib contributed several grade 3 toxicities, including pleural effusion, pancytopenia, and chronic bloody diarrhea (**Table 4**).

**Figure 3:**
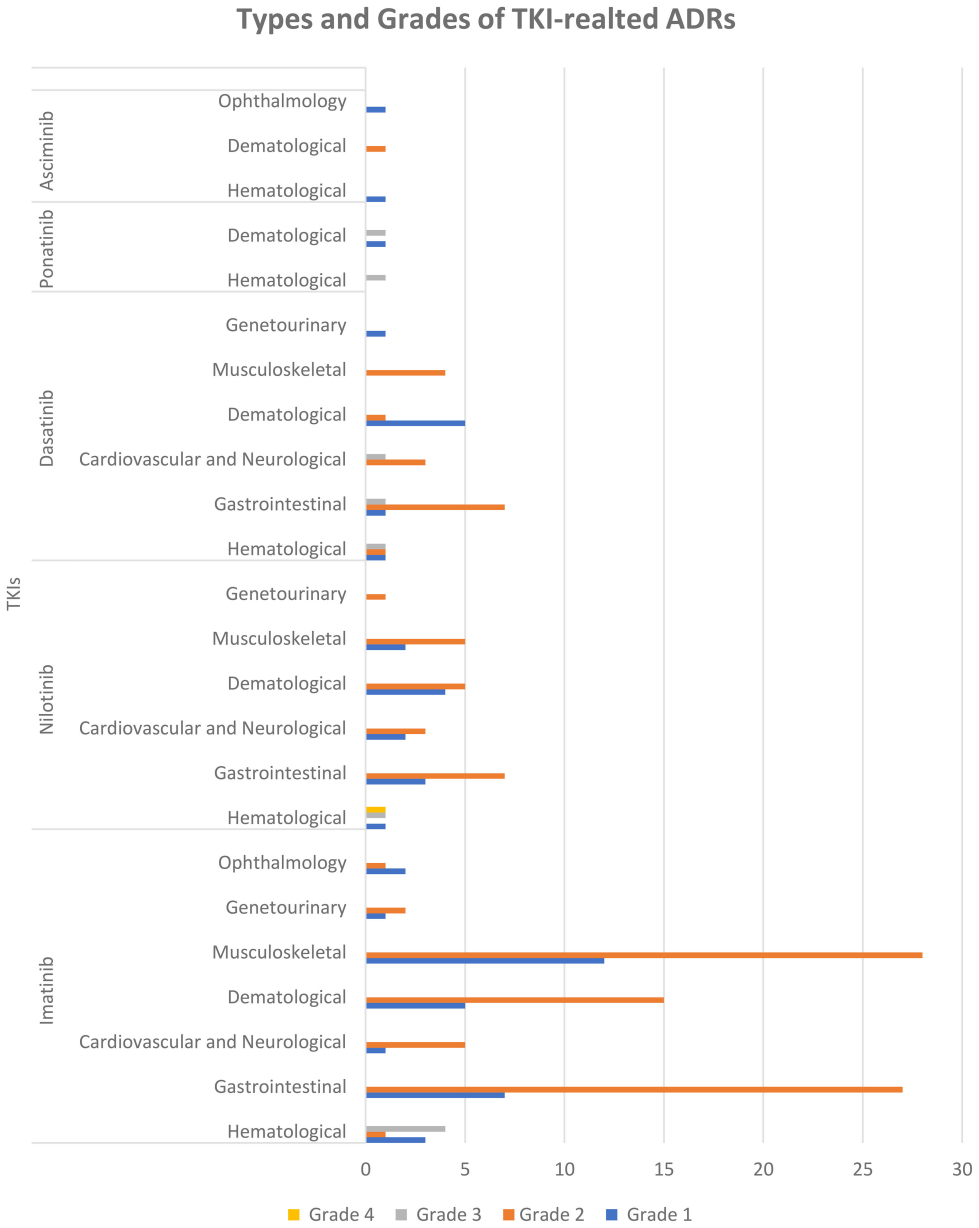
Distribution of adverse drug reaction categories and grades associated with tyrosine kinase inhibitors.

Ponatinib and asciminib each produced three ADRs, including two grade 3 events with ponatinib (severe generalized rash and cytopenia). Asciminib ADRs were primarily dermatologic and mild. An overview of types and grades of ADRs on all TKIs are shown in **Figure 3**.

Management of ADRs was largely supportive, with symptomatic treatments effectively controlling most grade 1–2 events, while hematologic and more severe toxicities such as pleural effusions required treatment interruption, dose modification, switching TKIs, or discontinuation in line with guideline-based practice. Overall, higher-grade events were the main drivers of therapy modification or discontinuation.

### 3.6 Treatment discontinuation

Across the cohort, loss of response or resistance was the most common reason for TKI discontinuation, followed by achievement of TFR and occurrence of ADRs. With imatinib, 56 of 107 patients (52.3%) stopped therapy. Among these, 24 discontinued due to resistance/loss of response, 7 due to ADRs, and 17 after achieving TFR. Nilotinib discontinuations occurred in 39% of 41 patients, mainly due to loss of response (6 patients), 1 ADR, and 4 TFR achievements. Notably, one patient progressed to advanced CML, necessitating discontinuation. Dasatinib was discontinued by 13 of 26 patients (50%), with 6 losing responses, 3 experiencing ADRs, and 2 achieving TFR. For ponatinib and asciminib, discontinuations were limited: two ponatinib patients and one asciminib patient were lost to follow-up, while one patient in each group discontinued due to ADRs.

To note, pregnancy-related discontinuation occurred in four female patients despite prior counseling. TKIs were immediately switched to interferon upon pregnancy confirmation. Three maintained MMR throughout pregnancy, while one lost MMR but regained it postpartum. One patient chose to continue breastfeeding despite recommendations. Finally, two patients (one on imatinib, one on nilotinib) discontinued TKIs due to undergoing hematopoietic stem cell transplantation (HSCT).

Disease progression was uncommon. During follow-up, two patients progressed from chronic phase CML to accelerated phase, while no patients progressed to blast crisis. Four deaths occurred during the study period. Causes of death were cardiac arrest (n=2), myocardial infraction (n=1) and diabetic ketoacidosis (n=1).

## 4 Discussion

CML is a rare disease in Oman, and local data on its management are limited due to small population size, low incidence, and the absence of a national registry. Our analysis, representing the first comprehensive evaluation of CML management in Oman, showed that imatinib was the most common first-line TKI, favored for its availability, likely cost-effectiveness, and safety, while nilotinib and dasatinib were less used due to access limitations. About half of patients achieved MMR on imatinib, with variable responses and ADRs across agents and doses. TFR was attempted in some patients, with outcomes comparable to international data despite resource constraints.

In our cohort of 112 patients, the mean age at diagnosis was 43.7 years, nearly a decade younger than that reported in Western series (56–57 years) (10). Similar findings have been described regionally, including Saudi Arabia, where the median age was 43.4 years (11). This younger age distribution may reflect population demographics, genetics, or environmental factors. While younger age often predicts better tolerance of long-term TKI therapy, several studies suggest it may also correlate with a higher risk of relapse following treatment discontinuation (12).

Unlike most Western studies that show male predominance (10), women represented the majority (56.3%) of patients in this study. This observation is clinically relevant: women of childbearing age face unique therapeutic challenges given the teratogenicity of all TKIs. Indeed, 5.4% of women in this series were pregnant during therapy, underscoring the importance of reproductive counseling and careful planning. Biological sex differences may also influence response, with men reported to have poorer molecular outcomes (13).

Nearly half of patients were overweight or obese. Previous research indicates that obesity can lower imatinib plasma concentrations and reduce molecular response rates, while non-obese patients may experience more AEs (14). Obesity also contributes to cardiovascular risk, particularly relevant for second-generation TKIs such as nilotinib (15).

Comorbidities were common, with 29.5% having a CCI of 1–2 and 16.1% ≥5. Higher CCI scores are associated with inferior survival and often influence clinicians to favor imatinib, given its safer long-term profile (16). Over half of patients presented with intermediate or high Sokal risk at diagnosis, reflecting the more advanced disease stage at presentation observed in real-world practice (17). Although newer TKIs may mitigate some high-risk features, their adoption in Oman remains limited by affordability and procurement constraints.

Imatinib remained the dominant first-line therapy in our cohort, prescribed in nearly 90% of patients due to its safety, affordability, and generic availability, a practice that contrasts with Western centers where second-generation TKIs such as nilotinib and dasatinib are increasingly favored, particularly in younger or higher-risk patients (18).

Almost half of imatinib-treated patients eventually required a switch, most often to nilotinib or dasatinib, mirroring global sequencing practices (19). Later-line therapies such as asciminib and ponatinib were used sparingly due to restricted access. Interestingly, first-line choice was not strongly associated with comorbidity burden, echoing findings by Ono (2021) (20), though some patients with cardiovascular disease still received nilotinib, contrary to ELN recommendations (18). These patterns highlight both resource-driven practice in Oman and the need for pharmacist-led stewardship to ensure comorbidity screening, guideline-based TKI selection, and structured monitoring to optimize outcomes.

Achieving MMR represents a critical therapeutic milestone in CML therapy, closely tied to long-term survival and reduced risk of progression. In our cohort, about half of patients reached MMR on first-line therapy, lower than the ~80% reported in the IRIS trial (21). The median time to MMR with imatinib was 21 months versus 18 months in IRIS, with one-third requiring more than three years to achieve response. The absence of early responders at three months likely reflects real-world constraints such as variable adherence, irregular molecular testing, and heterogeneous disease risk (22).

In our cohort, patients treated with nilotinib demonstrated shorter observed times to MMR than those treated with imatinib (median 5.5 vs. 21 months), consistent with its greater potency (23). Absolute MMR rates, however, were similar, suggesting comorbidities and baseline risk still influence outcomes. In the second-line setting, higher observed MMR rates were seen with nilotinib (79.2%) than with dasatinib (54.5%); however, these findings should be interpreted cautiously given the retrospective design and limited sample size. Ponatinib demonstrated meaningful clinical activity despite limited use (n=5). In contrast, imatinib rechallenge (n=8) and asciminib (n=3) showed minimal observed responses; however, interpretation is constrained by the very small sample sizes. As reported in prior studies, later-line responses were poor, reflecting diminishing benefit after multiple TKI failures (6). Attrition across lines was marked: 55% stayed on first-line, 29% moved to second-line, and fewer than 11% advanced beyond.

TFR was attempted in a proportion of patients. Patients discontinuing imatinib required longer exposure but maintained TFR beyond 36 months, exceeding the median in the STIM trial (Mahon et al., 2010). By contrast, second-generation TKIs were able to attempt TFR after shorter exposure durations and demonstrated numerically higher short-term TFR maintenance rates: 85.7% of patients stopping nilotinib maintained TFR at 48 weeks, compared with 51.6% in ENESTfreedom (16), and two-thirds of those stopping dasatinib remained in remission at six months, similar to the DADI trial (24). These results align with global data: imatinib generally requires prolonged treatment to support safe discontinuation, while nilotinib and dasatinib allow earlier and more successful TFR attempts.

The safety profile of TKIs in our cohort was broadly consistent with international reports. Most ADRs were mild to moderate and manageable with supportive care. Two cases of grade 3–4 hematological ADRs occurred with nilotinib, including anemia, thrombocytopenia, and neutropenia, in line with the findings of Cheng et al. (2023) (25). Dasatinib produced several grade 3 events, including pleural effusion and pancytopenia, managed through temporary discontinuation. Imatinib generally demonstrated the most favorable long-term safety and was often preferred after intolerance or serious toxicity with other agents, consistent with NCCN recommendations (26).

Treatment discontinuation occurred not only for toxicity or intolerance but also due to progression, unmet milestones, or as part of TFR attempts. In addition, pregnancy prompted discontinuation in some women. Current guidance recommends achieving deep molecular response before stopping therapy and using interferon-α as a safer alternative during gestation (27).

This study had several limitations, such as retrospective design, modest sample size and single-center data. Molecular outcomes were primarily evaluated up to major molecular response (MMR; BCR-ABL1 ≤0.1% IS). Although our center currently utilizes a quantitative BCR-ABL1 assay capable of detecting deep molecular responses (MR^4^ and MR^4.5^ or lower), this technology was introduced relatively recently. International guidelines, however, emphasize the importance of monitoring deep molecular responses *(MR^4 or MR^4.5)* for more comprehensive assessment of long-term disease control. In addition, during the research period, no standard BCR-ABL1 kinase domain mutation testing was available, including T315I mutation analysis. As result, we were unable to assess the influence of mutation status on TKI selection and treatment results. There were no reordered T315I mutation findings for ponatinib-treated individuals in this group. The extended study duration, covering over a decade of practice, may also introduce temporal variability—treatment access, monitoring strategies, and clinical decision-making have evolved substantially over this period. A shorter study window could have offered a more consistent view of current practice; however, the small national patient pool necessitated inclusion of a longer timeframe to ensure adequate sample size. Limited access to newer TKIs constrained evaluation of later-line therapies. Imatinib predominance reduced comparative power between agents, and small TFR numbers limit extrapolation. Furthermore, the relatively small sample sizes in several treatment subgroups limited feasibility of multivariable analyses, and findings should therefore be interpreted as observational associations. Nevertheless, strengths include conducting the study at a national hematology referral center, providing representative RWD for Oman. Comprehensive demographic, clinical, and treatment variables were captured, and management strategies were largely aligned with international guidelines. Structured follow-up and patient counseling further strengthen the study, underscoring the value of multidisciplinary and pharmacist involvement in optimizing outcomes.

## 5 Conclusion

This first national study of CML in Oman shows that while imatinib remains the predominant frontline agent due to accessibility, it was associated with slower and less frequent achievement of molecular responses compared with second-generation TKIs. These findings underscore the importance of a response-guided treatment strategy, where early identification of suboptimal responders and timely reassessment of response and consideration of alternative TKIs in appropriate patients may help optimize long-term outcome. Real-world outcomes demonstrated modest MMR rates compared with clinical trials, but TFR was achievable and consistent with international experience. Safety patterns aligned with global data, and treatment sequencing strategies mirrored higher-resource settings despite limited drug availability. Overall, optimizing CML outcomes in Oman will require broader access to second-generation TKIs and the establishment of a national CML registry to support data-driven care. These measures will be essential to align local practice with evolving international standards and to maximize the long-term benefits of TKI therapy.

## Data Availability

The raw data supporting the conclusions of this article will be made available by the authors, without undue reservation.
